# A randomized phase II study of acyclovir for the prevention of chemotherapy-induced oral mucositis in patients undergoing autologous hematopoietic stem cell transplantation

**DOI:** 10.1186/s12903-023-03623-6

**Published:** 2023-12-15

**Authors:** Junshik Hong, Hee-Kyung Park, Sung-Ho Chang, Ja Min Byun, Dong-Yeop Shin, Youngil Koh, Sung-Soo Yoon, Youngnim Choi, Inho Kim

**Affiliations:** 1 Department of Internal Medicine, Seoul National University Hospital, Seoul National University College of Medicine, 101 Daehak-ro, Jongno-gu, Seoul, 03080 Korea; 2 Cancer Research Institute, Seoul National University, Seoul, Korea; 3 Department of Oral Medicine and Oral Diagnosis, Seoul National University Dental Hospital, Seoul National University School of Dentistry, Seoul, Korea; 4 Department of Immunology and Molecular Microbiology, School of Dentistry and Dental Research Institute, Seoul National University, 101 Daehak-ro, Jongno-gu, Seoul, 03080 Korea

**Keywords:** Oral mucositis, Herpes simplex virus, Autologous stem cell transplantation, Acyclovir, Chemotherapy

## Abstract

**Objectives:**

To prove our hypothesis that acyclovir prophylaxis in autologous hematopoietic stem cell transplantation (AHSCT) recipients with hematologic malignancies (HM) reduces the incidence of chemotherapy-induced oral mucositis (CIOM) by inhibiting the intraoral HSV reactivation during the neutropenic period, we conducted a randomized phase II study of acyclovir for the prevention of CIOM in adult HSV sero-positive AHSCT recipients.

**Methods:**

Patients were randomized to either the study group (acyclovir 400 mg PO bid until neutrophil engraftment) or the control group (no prophylaxis) and received AHSCT. Oral examination and sampling for HSV were performed at three timepoints of AHSCT.

**Results:**

In 54 patients who were randomized (for intention-to-analysis), the incidence of CIOM was 16.0% (4/25 patients) and 58.6% (17/29 patients) in the study group and the control group, respectively (P = 0.001). In 49 patients who completed the study (for per-protocol analysis), the incidence of CIOM was 13.0% (3/23 patients) and 61.5% (16/26 patients) in the study group and the control group, respectively (P = 0.001). In addition, HSV-1 PCR positivity in the study group was significantly lower than that the control group (4.3% vs. 46.2%, P = 0.001). A strong association between the HSV-1 reactivation status and CIOM was reconfirmed.

**Conclusions:**

Prophylactic use of oral acyclovir effectively reduced the incidence of CIOM in patients with HM who were undergoing AHSCT.

**Trial registrations:**

This trial was registered at the Clinical Research Information Service in the Republic of Korea under the number KCT0003885 (registration date 03/05/2019).

**Supplementary Information:**

The online version contains supplementary material available at 10.1186/s12903-023-03623-6.

## Backgrounds

Chemotherapy-induced oral mucositis (CIOM) is a significant side effect of chemotherapy in cancer patients. A significant correlation was found between a 1-point increase in the oral mucositis score and several adverse outcomes, including more days with fever, parenteral nutrition, hospitalization, and higher mortality at 100 days in patients undergoing chemotherapy [1]. Delayed regeneration of intraoral epithelium damaged by anticancer drugs is the basic mechanism of CIOM, but it is expected that there are additional contributors to its development and exacerbation given that it does not occur to the same extent in all patients. Oral resident flora or oral infections caused by bacteria, viruses, and Candida before treatment may cause or worsen CIOM after chemotherapy. In these cases, CIOM is often improved by empirical antimicrobial treatment [2]. In our previous collaborative study by the Seoul National University College of Medicine and Seoul National University School of Dentistry, we showed that herpes simplex virus (HSV) reactivation was independently associated with the increased incidence of CIOM in patients with hematologic malignancies (HMs) and clinical symptoms estimated by the Oral Mucositis Daily Questionnaire (OMDQ) were significantly associated with CIOM and HSV-1 status [3]. This suggests that HSV plays a considerable role in the development of CIOM in present-day chemotherapy among patients with hematologic malignancies. Types of hematologic malignancies (acute leukemia or myelodysplastic syndrome) and male sex were independent risk factors for CIOM along with HSV-1 positivity [3].

The Center for International Blood and Marrow Transplant (CIBMTR) guidelines for the prevention and treatment of infections in hematopoietic stem cell transplantation (HSCT) recipients clearly and strongly recommend the use of the antiviral agent acyclovir to HSV-seropositive allogeneic HSCT recipients during the preengraftment period to prevent HSV reactivation based on good quality of evidence (level AI from the evidence-based rating system) [4]. However, for autologous HSCT recipients, the guideline mentions that antiviral prophylaxis can be applied if an HSV-seropositive patient has a significant risk of developing HSV infection, including CIOM (level CIII) [4]. One exception is that it is recommended to use acyclovir for more than 30 days after autologous HSCT if a patient has undergone repeated HSV infection (level BIII) [4]. This is because there has been no clinical study that prospectively evaluated whether acyclovir prophylaxis is beneficial to autologous HSCT recipients. Actually, in many centers, autologous HSCT recipients receive prophylactic acyclovir with the assumption that the sequelae of conditioning therapy are generally comparable to that of allogeneic HSCT. However, it has never been evaluated whether prophylactic use of acyclovir is really necessary equally in autologous HSCT setting where mortality is almost negligible and there is no need of immunosuppressant use during the procedure.

Based on this background and the results of our previous work [3], and to make solid evidence for dental clinical evidence, we conducted a randomized phase II study of acyclovir for the prevention of CIOM in HSV-seropositive patients undergoing autologous HSCT with a hypothesis that acyclovir prophylaxis in patients with lymphomas and multiple myeloma (MM) undergoing autologous HSCT would inhibit intraoral reactivation of HSV, thereby reducing the incidence of CIOM.

## Methods

### Patients

The current study included patients who (1) were aged 19 years or older; (2) were diagnosed with malignant lymphoma or MM and were undergoing autologous HSCT at Seoul National University Hospital; and (3) were negative for HSV IgM and positive for HSV IgG within one month before the initiation of autologous HSCT. Patients were excluded if they (1) had previously experienced Herpesviridae reactivation two more times or any recent Herpesviridae reactivation during treatment of HM before HSCT; (2) already had an oral ulcer, HSV polymerase chain reaction (PCR)-positivity of oral sample, severe dental diseases, or poor oral hygiene at the time of screening oral examination; (3) prophylactic acyclovir was either mandatory or contraindicated according to the investigators’ judgment for any other reason; (4) had a glomerulus filtration rate of less than 60 mL/min/1.73 m^2^ according to the Modification of Diet in Renal Disease methods; and (5) had a history of hypersensitivity to acyclovir or valacyclovir.

### Screening and randomization

The eligibility of all autologous HSCT recipients newly admitted to HSCT ward was pre-screened by the researchers. Patients who were potentially eligible were recommended to participate in the current study. A signed consent form was completed by patients were willing to participate in the current study. Patients were screened for HSV IgG and IgM status and history of past viral infection for HSV, varicella-zoster virus (VZV), and cytomegalovirus (CMV). Patients who were finally confirmed eligible were subsequently randomized in a 1:1 fashion to either the study group (acyclovir prophylaxis group) or the control group (no prophylaxis). Randomization was stratified by HM (MM vs. lymphoma) and sex (male vs. female) to make even distribution of underlying HM and because male sex was an independent risk factor of CIOM in our previous study [3].

### Autologous hematopoietic stem cell transplantation

All patients were admitted to the HSCT ward at Seoul National University Hospital. Ciprofloxacin 500 mg *per os* bid and intravenous micafungin 50 mg once daily were uniformly applied for antibacterial and antifungal prophylaxis, respectively, from the first date of conditioning to the date of neutrophil engraftment (defined by absolute neutrophil count ≧ 1,000/µL for 3 consecutive days). Brushing with a soft toothbrush and gargling with sodium bicarbonate solution and nystatin were encouraged. Several different kinds of multiagent conditioning regimens were used for patients with lymphoma (Table S1). For most MM patients, busulfan 3.2 mg/kg for 3 days (D-6 to D-4) in combination with melphalan 70 mg/m^2^ for 2 days (D-3, -2) was used for conditioning (Table S1).

### Intervention

In principle, acyclovir was administered orally. Patients in the study group took acyclovir 400 mg *per os* bid from the first date of conditioning to the date of neutrophil engraftment. If oral medications became intolerant during HSCT due to severe CIOM or any other cause, they could be transiently changed to intravenous acyclovir 250 mg/m^2^ bid, to sustain the prophylactic effects of acyclovir. In the control group, autologous HSCT was performed without acyclovir or any other antiviral prophylaxis. However, if a patient showed suspicious symptoms of CIOM or other infections caused by HSV reactivation or other viral infections, such as CMV, during the transplant procedure, appropriate antiviral agents were administered for therapeutic purposes, as decided by investigators.

### Oral examination, sampling, and evaluation

Oral examination and sampling were performed by a dentist (H-K Park) and research nurses who were trained and supervised by the dentist at the following three timepoints: (1) baseline – within + 2 days from the first day of conditioning, (2) during HSCT – at day + 8 (± 2) from the date of stem cell infusion (D0). If a patient complained of oral pain, oral examination and sampling were conducted within two days, and those results were substituted for the results of the day + 8 timepoint, and (3) at the end of autologous HSCT – within 7 days from the date of neutrophil engraftment.

The presence and severity of CIOM are described using the WHO Toxicity Criteria – Oral mucositis scoring system scale (Table S2).

To obtain samples for HSV-1 and HSV-2 reactivation in the oral mucosa, the oral mucosa was brushed with a sterile 30 mm × 30 mm Imobilon®-P Transfer Membrane (Merck Millipore, Billerica, MA, USA) for 30 s. If there was an intraoral lesion, the area was swabbed. If there was no intraoral lesion, the buccal mucosa was swabbed. DNA was separated from the collection membrane using the PowerSoil® DNA Isolation Kit (MOBIO Laboratories, Carlsbad, CA, USA) and diluted in a 100 mL buffer solution. PCR was performed using an HSV 1/2 PCR kit (BioCore, Geumcheon-gu, Seoul, Korea) to detect HSV-1 and HSV-2.

### Study endpoints and statistical analysis

The primary endpoint was the incidence of CIOM during autologous HSCT according to acyclovir prophylaxis. The secondary endpoint was the incidence of HSV reactivation during autologous HSCT according to acyclovir prophylaxis and the association between oral HSV reactivation and CIOM. The intent-to-treat analysis was planned for all randomized patients. The per-protocol analysis was planned for subjects who completed the study without a protocol violation.

Considering the results of previous studies [5, 6], the expected CIOM incidence rate for autologous HSCT recipients was approximately 60%. Assuming that acyclovir prophylaxis induces a 25% reduction in CIOM incidence (p0 = 0.35, p1 = 0.60), the number of patients required in Fleming’s one-stage phase II study design for α = 0.05, power (1-β) = 0.8 was 24. Considering the 10% dropout rate, 27 patients for each group and a total of 54 patients needed to be randomized. This study was planned as a randomized clinical trial that paralleled two phase II studies for each arm and indirectly compared the differences in the incidence of CIOM.

Demographic data are expressed as the mean, median, standard deviation, maximum, and minimum for continuous variables, and the frequency and ratio are summarized for categorical variables. Analyses were primarily performed for all subjects randomized in the study, but additional per-protocol analyses were also conducted from subjects who fully complied with the intervention. The association between two independent variables was analyzed through Fisher’s exact test or the chi-square test, where appropriate. Continuous variables were analyzed by t test or Wilcoxon’s signed rank test paired according to normality assumption satisfaction, and categorical variables were analyzed by McNemar test.

## Results

### Patients

The current study was registered to clinicaltrials.gov before the initiation of patient enrollment. From June 2019 to July 2022, a total of 54 patients were randomized, 25 patients for the study group and 29 patients for the control group. After the completion of autologous HSCT of the last enrolled patients, data were analyzed.

**Fig. 1:**
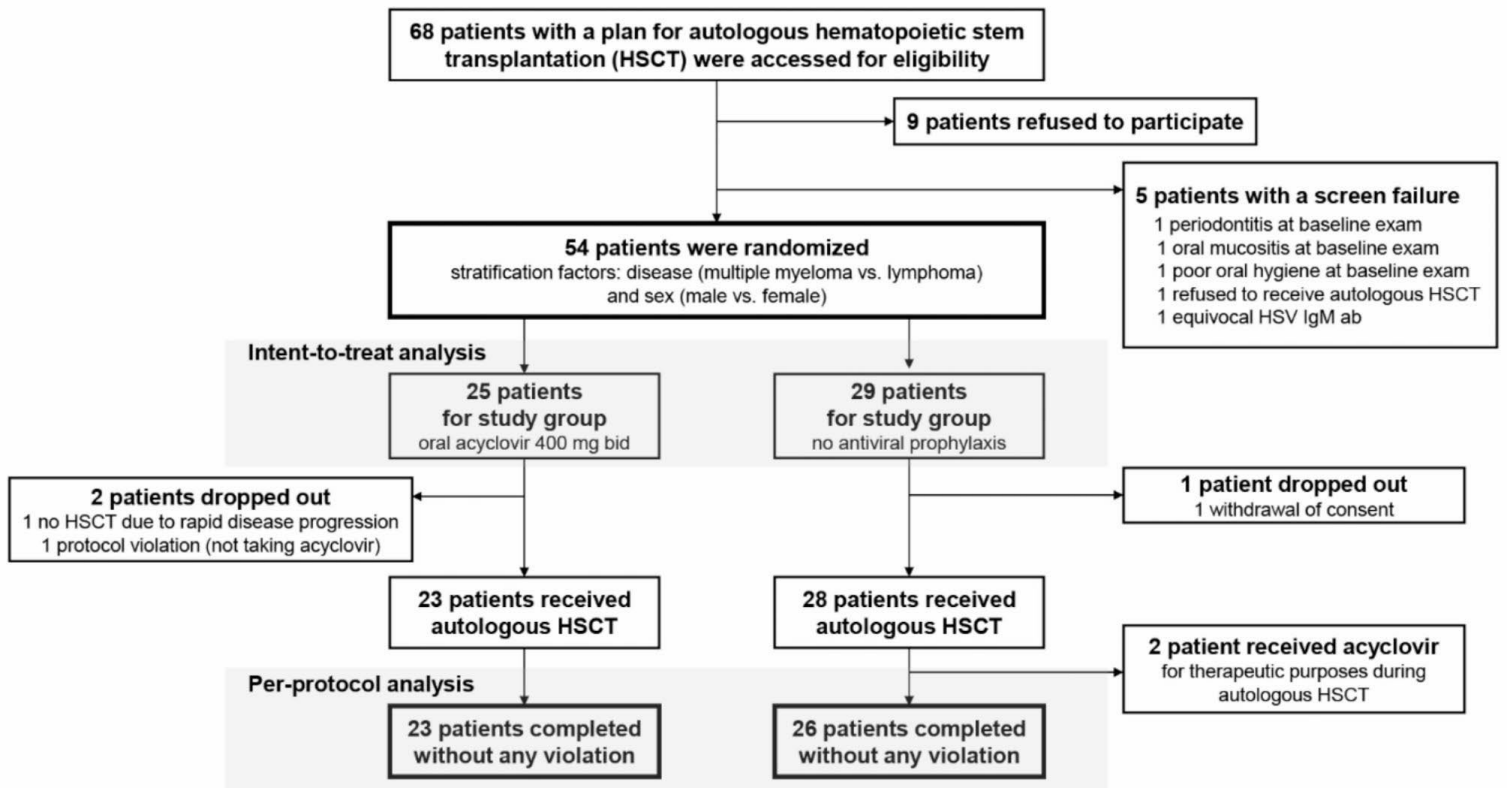
A CONSORT diagram

All randomized patients were negative for HSV IgM and positive for HSV IgG. Three patients dropped out after randomization and before treatment initiation. One patient from the control group withdrew consent before the initiation of the HSCT procedure. Another patient from the study group experienced rapid disease progression while waiting for admission; thus, autologous HSCT was cancelled. The other patient was allocated to the study group but did not take acyclovir tablets during the HSCT period due to a communication error. In addition, two patients who were allocated to the control group received acyclovir before neutrophil engraftment for empirical therapeutic purposes due to pharyngitis and fever. Thus, the per-protocol analysis was conducted using 49 patients who completed autologous HSCT without any violation (Fig. 1). The application of the two stratification factors in the computerized randomization system resulted in a slight imbalance of the patient numbers between the two groups (25 vs. 29 patients), but baseline patient characteristics were balanced between the two groups (Table 1).

**Table 1:** Patient characteristics

	Both groups	Study group	Control group	P
N	54	25	29	
**Age (years)**				0.709
Median (range)	56 (19–69)	54 (19–66)	57 (23–68)	
**Sex**				0.951
Male	30	14	16	
Female	24	11	13	
**Types of disease**				0.651
Lymphoma	32	14	18	
Multiple myeloma	22	11	11	
**Disease status before HSCT**				0.799
First complete remission (CR1)	22	11	11	
CR ≧ 2	13	6	7	
Partial remission (PR)	18	8	10	
Others	1	0	1	
**CD34 + cells infused (x10** ^**6**^ **/kg)**				0.766
Mean (standard deviation)	5.45	5.32	5.55	
**Best Response to HSCT**				0.332
CR	45	23	22	
PR	6	2	4	
Stable disease (SD)	1	0	1	
Disease progression (PD)	2	0	0	
**Relapse or PD after remission**				0.198
Yes	13	4	9	
No	41	21	20	

### HSV and CIOM outcomes

Among 54 randomized patients, the incidence of CIOM was 16.0% (4 out of 25 patients) and 58.6% (17 out of 29 patients) in the study group and the control group, respectively (P = 0.001; Table 2). Two out of 25 patients (8%) randomized to the study group had a positive HSV-1 PCR result during autologous HSCT. In contrast, 12 patients (41.4%) out of 29 patients allocated to the control group became HSV-1 PCR positive during autologous HSCT (P = 0.005). In the per-protocol analysis of the 49 patients, the incidence of CIOM was 13.0% (3 out of 23 patients) and 61.5% (16 out of 26 patients) in the study group and the control group, respectively (P = 0.001; Table 3). HSV-1 PCR positivity was reported in 4.3% (one out of 23 patients) of the study group and 46.2% (12 out of 26 patients) of the control group (P = 0.001). There was no HSV-2 reactivation among all subjects. All patients who experienced CIOM in the study group had grade 1 CIOM, while 3 patients in the control group had grade 2 CIOM according to the WHO scale. These results suggest that acyclovir prophylaxis during autologous HSCT significantly reduced the incidence of CIOM and HSV-1 reactivation in the oral cavity.

**Table 2:** Herpes simplex virus reactivation and chemotherapy-induced oral mucositis outcomes: Intent-to-treat analysis (N = 54)

	Both groups	Study group	Control group	P
N	54	25	29	
**CIOM (by WHO scale)**				
Yes ( ≧ 1)	21 (38.9%)	4 (16.0%)	17 (58.6%)	0.001
Grade 0 (No CIOM)	29	20	9	
Grade 1	18	4	14	
Grade 2	3	0	3	
Grade 3	0	0	0	
Grade 4	0	0	0	
Not Evaluated	4	1	3	
**HSV-1 PCR**				
Positive at day + 8	12	2	10	0.019
Positive at the end of HSCT	10	1	9	0.013
Positive at any of the 2 timepoints	14 (25.9%)	2 (8.0%)	12 (41.4%)	0.005

**Table 3:** Herpes simplex virus reactivation and chemotherapy-induced oral mucositis outcomes: Per-protocol analysis (N = 49)

	Both groups	Study group	Control group	P
N	49	23	26	
**CIOM by WHO scale**				
Yes ( ≧ 1)	19 (38.8%)	3 (13.0%)	16 (61.5%)	0.001
Grade 0 (No CIOM)	30	20	10	
Grade 1	16	3	13	
Grade 2	3	0	3	
Grade 3	0	0	0	
Grade 4	0	0	0	
**HSV-1 PCR**				
Positive at day + 8	11	1	10	
Positive at the end of HSCT	9	0	9	
Positive at any of the 2 timepoints	13 (26.5%)	1 (4.3%)	12 (46.2%)	0.001

### The association between oral HSV-1 reactivation and CIOM

Among the per-protocol population (N = 49), we investigated the association between HSV-1 reactivation and the occurrence of CIOM during autologous HSCT (Table 4). Regardless of acyclovir prophylaxis, HSV-1 reactivation status was significantly associated with the incidence of CIOM (P < 0.0001). Among the 13 patients who had oral HSV-1 reactivation, 12 patients developed CIOM (92.3%). Interestingly, only 1 patient (8.3%) in the study group had HSV-1^+^CIOM^+^ (%), while 11 patients in the control group (91.7%) did. In contrast, among the 36 patients who did not have oral HSV-1 reactivation, 7 patients (19.4%) experienced CIOM during autologous HSCT. Specifically, among the 7 HSV-1^−^CIOM^+^ patients, only 2 patients belonged to the study group. These results show that the association between oral HSV-1 reactivation and CIOM is reconfirmed.

**Table 4:** Association between HSV-1 reactivation and the development of chemotherapy-induced oral mucositis (N = 49)

	Both groups	Study group	Control group
N	49	23	26
HSV(+) and CIOM (+)	12^*^	1	11
HSV (+) and CIOM (-)	1^*^	0	1
HSV (-) and CIOM (+)	7^*^	2	5
HSV (-) and CIOM (-)	29^*^	20	9

*P < 0.0001 by Chi-square test

Because only two patients showed later HSV-1 PCR positivity (i.e., not at day + 8 but at the end of HSCT), the association of the onset of HSV with the incidence or severity of CIOM could not be analyzed.

## Discussion

Our study clearly and concisely shows that acyclovir prophylaxis effectively reduces the incidence of HSV-1 reactivation and CIOM in HSV-seropositive autologous HSCT recipients during the neutropenic phase.

Earlier studies in the 1990s mostly reported that oral HSV reactivation was not a major etiologic factor for CIOM [7–9]. In contrast, more recent studies have suggested a potential connection between HSV and CIOM [2, 10, 11]. In our previous work, we demonstrated that HSV-1 reactivation was an independent factor affecting the incidence of CIOM in patients with HM who were undergoing chemotherapy or HSCT [3, 12]. More frequent antimicrobial prophylaxis could be the reason for the difference; currently, the use of prophylactic antibiotics and antifungal agents has become almost routine in HSCT [13, 14] and is supported in some settings of intensive chemotherapy with evidence [15]. Thus, it is more difficult for bacteria or fungi in the oral cavity to cause or contribute to the development of CIOM. Instead, HSV could be a major causative microorganism for CIOM. This is supported by our study in which all patients received prophylactic ciprofloxacin and micafungin during autologous HSCT procedures.

HSCT recipients have the highest incidence of CIOM among patients with HM [16, 17]. Approximately half of autologous HSCT recipients experience CIOM; one study showed that 64.3% of autologous HSCT recipients underwent CIOM among 115 patients with MM [6]. Another study reported a 53.7% CIOM incidence among autologous HSCT recipients [5]. In our previous work, 12 out of 23 HM patients (52.2%) who received autologous HSCT developed CIOM [3]. The CIOM incidence in the control group of the current study coincides with those reported previously. These high incidences suggest that CIOM needs to be actively managed in autologous HSCT recipients, thus supporting the role of acyclovir prophylaxis.

We are aware of some limitations in our study. First, we used an open-label design. We chose not to employ blinding for two specific reasons: first, to facilitate patient enrollment. Second, to enhance the comfort of patients in the control group. We though that being aware of their allocation to the control group could potentially help prevent any unnecessary delays in the therapeutic (i.e., not prophylactic) use of acyclovir if needed. However, we cannot deny that blinding of treatment groups by using placebo would have made the results even stronger. Second, some may argue that if many centers are already doing acyclovir prophylaxis in autologous HSCT recipients, the impact of this study is lessened. However, our study clearly showed HSV as a predominant factor for CIOM in modern HSCT equipped with strong anti-bacterial and fungal prophylaxis as well as adding solid evidence for clinical practice. Finally, we selected 400 mg *per os* bid (800 mg/day) as a prophylactic dose of acyclovir with reference to a recent guideline [18] and the EBMT guideline, which mentions 600 (200 mg tid) to 1,600 mg/day (800 mg bid) of oral acyclovir [19]. However, the optimal dose and role of lower doses of acyclovir remain to be defined. In order to balance the two groups for the stratification factors, the randomization was not perfectly even and there is a slight shortage of patient number in the study group. However, substantial difference of CIOM incidence and HSV reactivation was already present between the two groups, thus it does not alter this study’s conclusion at all. We did not include analyses with patient-reported questionnaires focused on subjective feelings of oral soreness or discomfort on a daily basis. Nevertheless, our study obviously demonstrates the usefulness of acyclovir in preventing CIOM in autologous HSCT recipients.

## Conclusions

In conclusion, prophylactic use of oral acyclovir effectively reduced the incidence of CIOM in patients with HM who were undergoing autologous HSCT during the neutropenic phase.

### Electronic supplementary material

Below is the link to the electronic supplementary material.

Supplementary Material 1

## Data Availability

The dataset used and/or analyzed during the current study is available from the corresponding authors on reasonable request.

## Abbreviations

CIOM: Chemotherapy-induced oral mucositis
HSV: herpes simplex virus
HM: hematologic malignancy
HSCT: hematopoietic stem cell transplantation
MM: multiple myeloma
PCR: polymerase chain reaction
VZV: varicella-zoster virus
CMV: cytomegalovirus
