# Supplementary material for: A randomized phase II study of acyclovir for the prevention of chemotherapy-induced oral mucositis in patients undergoing autologous hematopoietic stem cell transplantation
Source: BMC Oral Health. 2023 Dec 15;23:1008. doi: 10.1186/s12903-023-03623-6 (PMC10724996; doi:10.1186/s12903-023-03623-6)
Supplement: Supplementary file 1 — Supplementary Material 1 [file 12903_2023_3623_MOESM1_ESM.docx]

**Supplementary Material for online publication**

**Table S1**. Conditioning regimens used.

| Lymphoma (N = 32) | Regimen | Patients received |
| --- | --- | --- |
| BEAM | BCNU 300 mg/m^2^ qd, D-7  Etoposide 100 mg/m^2^ bid, D-6 to D-3  Ara-C 100 mg/m^2^ bid, D-6 to D-3  Melphalan 140 mg/m^2^ qd, D-2 | 3 |
| BuEAM | Busulfan 3.2 mg/kg qd, D-6 to D-5  Etoposide 200 mg/m^2^ bid, D-4 to D-3  Cytarabine 1g/m^2^ qd, D-4 to d-3  Melphalan 140 mg/m^2^ qd, D-2 | 4 |
| BuCyEto | Busulfan 3.2 mg/kg qd, D-7 to D-5  Etoposide 400 mg/m^2^ bid, D-5 to D-4  Cyclophosphamide 50 mg/kg qd, D-3 to D-2 | 15 |
| BuMelEto | Busulfan 3.2 mg/kg qd, D-7 to D-5  Etoposide 400 mg/m^2^ bid, D-5 to D-4  Melphalan 50 mg/m^2^ qd, D-3 to D-2 | 1 |
| ThioBuCy | Thiotepa 200 mg/m^2^ qd, D-8 to D-6  Busulfan 3.2 mg/kg qd, D-5 to D-4  Cyclophosphamide 60 mg/kg qd, D-3 to D-2 | 8 |
| Could not done |  | 1 |
| Multiple myeloma (N = 22) |  |  |
| BuMel | Busulfan 3.2 mg/kg qd, D-6 to D-4  Melphalan 70 mg/m^2^ qd, D-3 to D-2 | 21 |
| High dose Melphalan | Melphalan 100 mg/m^2^ qd D-3 to D-2 | 1 |

HSCT, hematopoietic stem cell transplantation

**Table S2**. The World Health Organization (WHO) Toxicity Criteria; Oral Mucositis Scoring System Scale

| Grade 0 | Grade 1 | Grade 2 | Grade 3 | Grade 4 |
| --- | --- | --- | --- | --- |
| Normal | Erythema and soreness | Ulceration, but can eat solid foods | Ulceration, diet limited to liquids | Ulceration of such severity that patient requires parenteral feeding |
